# Old Times

**Published:** 1874-09

**Authors:** 


### OLD TIMES.

WHO does not remember in the times gone by, half a hundred years or more ago, that if the fire went out in the kitchen during the night, as it sometimes would, especially if it happened to snow two or three feet, the nearest neighbor had to be visited to get a coal or two to kindle it at home ! ! But the little friction match has come into our dwellings, and is now considered an indispensable article in every household ; and yet it was unknown fifty years ago. But one day, a plodding old Dutchman, named Brandt, who wanted to take a short cut to fortune was trying to find out some way to turn silver into gold ; he took it into his head that it could be done by boiling and concentrating urine, and thus obtaining a solvent which would accomplish the desired result. He found a waxy residue that would take fire as easily as would alcohol by the application of flame ; in fact it would take fire if warmed, even by heating it in the hands ; this was phosphorus, one ounce only from one hundred gallons of the liquid. He sold his secret for two hundred dollars ; but this ounce was worth two hundred dollars. But nearly two hundred years passed away before anyone thought out a plan for kindling a fire with phosphorus. Up to 1834 a box holding fifty matches sold for two dollars, but then it was ascertained that they could be made of as inexpensive material as phosphorus, which could then be had for a trifle.

For some years the common friction match was known as a Loco Foco, which name was transferred to the Democratic Party in connection with a disorderly political meeting, some one putting out the lights, when one of the members having a match in his pocket u[illegible]d it to re-light the apartment. Dr. Nichol's, in his very valuable "Boston Journal of Chemistry," says that the first matches were made of very expensive materials, but it was found that if chloride of potash and sulphide of antimony were made into a paste and dried and subjected to friction, a light would be produced; so for convenience a bit of dry wood was dipped into the mixture, and when dry, it was dipped into melted brimstone, which, on being allowed to dry, made the common lucifer match, but for some years the smell of brimstone was a great objection to its use. In 1834 it occurred to an English chemist to dip a pine splinter into sulphur, then into a mixture of phosphorus, made into a paste with glue, a little sand, and red ochre; this is now the most common match, but there are several other kinds, one of which, the "Safety," will not ignite unless it is rubbed against a material which is attached to the box holding the matches.

Suffocation and other dangerous results have taken place from drawing in the breath at the instant of lighting a sulphur match; the instant any kind of match kindles, it should be held at arm's length from the body, to allow time for the fumes to rise above the head. There is a sweetness to the taste of some matches, and young children have been tempted to bite off the match end and eat it, with fatal results.

A convenient rat poison is made by throwing a few matches into melted grease, stir it up, place it in a saucer in the middle of the floor for the rats and mice and snakes to eat, which they will freely do to their own destruction.

But phosphorus will take fire of itself unless kept under water; to prevent then the match from setting itself on fire, it is covered with a thin film of gelatine, which perfectly excludes the air, so that now the match must be rubbed against something rough, to scale off this film and allow the air to get to the more inflammable substance underneath. The chemist is still alive who made the first phosphorus for matches at twenty-five hundred dollars a pound. It now sells in New York for a dollar a pound, obtained from bones; it requires a hundred pounds of coal to make one pound of phosphorus; requiring such a heat as to rapidly destroy the apparatus used in making it, while the inhalation of the fumes by those who make the matches produces in some constitutions dreadful ulcerations about the face and other parts of the system, while others seem to escape all injuries. There is a kind of rock in South Carolina discovered lately which contains phosphorus enough, in its crude state, to make matches sufficient to supply the world for a million of years.

